# Folic Acid-Conjugated Magnetic Oleoyl-Chitosan Nanoparticles for Controlled Release of Doxorubicin in Cancer Therapy

**DOI:** 10.3390/nano15060415

**Published:** 2025-03-07

**Authors:** Banendu Sunder Dash, Yi-Chian Lai, Jyh-Ping Chen

**Affiliations:** 1 Department of Chemical and Materials Engineering, Chang Gung University, Kwei-San, Taoyuan 33302, Taiwan; d000018157@cgu.edu.tw (B.S.D.);; 2 Department of Neurosurgery, Chang Gung Memorial Hospital at Linkou, Kwei-San, Taoyuan 33305, Taiwan; 3 Research Center for Food and Cosmetic Safety, College of Human Ecology, Chang Gung University of Science and Technology, Taoyuan 33305, Taiwan; 4 Department of Materials Engineering, Ming Chi University of Technology, Tai-Shan, New Taipei City 24301, Taiwan

**Keywords:** chitosan, oleic acid, folic acid, iron oxide, magnetic nanoparticles, doxorubicin

## Abstract

To develop an efficient drug delivery system, we co-entrapped superparamagnetic Fe_3_O_4_ and the chemotherapeutic drug doxorubicin (DOX) in oleoyl-chitosan (OC) to prepare DOX-entrapped magnetic OC (DOX-MOC) nanoparticles (NPs) through ionic gelation of OC with sodium tripolyphosphate (TPP). The NPs provide magnetically targeted delivery of DOX in cancer therapy. Using folic acid (FA)-grafted OC, FA-conjugated DOX-entrapped magnetic OC (FA-DOX-MOC) NPs were prepared similarly for FA-mediated active targeting of cancer cells with overexpressed folate receptors. Considering DOX loading and release, the best conditions for preparing DOX-MOC NPs were an OC:TPP mass ratio = 1:4 and OC concentration = 0.2%. These spherical NPs had a particle size of ~250 nm, 87.9% Fe_3_O_4_ content, 53.1 emu/g saturation magnetization, 83.1% drug encapsulation efficacy, and 2.81% drug loading efficiency. FA did not significantly change the physico-chemical characteristics of FA-DOX-MOC compared to DOX-MOC, and both NPs showed pH-dependent drug release behaviors, with much faster release of DOX at acidic pH values found in endosomes. However, FA could enhance the intracellular uptake of the NPs and DOX accumulation in the nucleus. This active targeting effect led to significantly higher cytotoxicity towards U87 cancer cells. These results suggest that FA-DOX-MOC NPs can efficiently deliver DOX for controlled drug release in cancer therapy.

## 1. Introduction

Inorganic nanoparticles may be incorporated into chitosan (CS) nanoparticles (NPs) intended for biological uses during synthesis. One example is the combination of superparamagnetic Fe_3_O_4_ with CS to fabricate drug-loaded magnetic nanocarriers for magnetic targeted drug delivery [1]. Using a permanent magnet for magnetic guidance following the injection of such nanocomposites, drug delivery to the tumor can be greatly improved via magnetic targeting. This can maximize the treatment efficacy while lowering systemic toxicity. Compared with traditional cancer treatment methods, this approach can increase drug availability to the tumor to elicit higher cytotoxicity towards cancer cells without killing cells in healthy tissues [2]. Chemotherapeutic drugs can be released by changing the pH value in an acidic endosomal microenvironment after endocytosis to enhance cytotoxicity towards cancer cells [3]. By incorporating Fe_3_O_4_ in smart drug-loaded NPs, a magnetic targeted drug delivery system could be designed. Under an external magnetic field, the magnetic NPs can direct the drug carrier to the treatment site, followed by controlled drug release in the low-pH endosomal environment, thus eliciting higher cytotoxicity toward cancer cells compared to free drugs [4]. To maximize the targeting effects of drug-loaded NPs, the surfaces of smart NPs can be modified with a ligand molecule, such as folic acid (FA), which can be recognized by folate receptors overexpressed on the cancer cell surface to improve the efficacy of drug delivery and exert higher cytotoxicity towards cancer cells [5]. This can not only improve the therapeutic effect but also reduce systemic side effects [6].

Doxorubicin (DOX) belongs to the anthracycline group of chemotherapeutic agents derived from *Streptomyces peucetius* and is widely employed in cancer treatment. Due to its molecular size, DOX can fit in spaces between base pairs in a DNA molecule. After being chelated into a DNA molecule, DOX can limit the DNA replication of cancer cells and function as a chemotherapeutic drug for cancer therapy [7]. As it is a nonspecific anticancer medication that targets DNA molecules during the cell cycle, it can be loaded into various NPs for cancer treatment. Biodegradable CS is often used as a delivery system to control drug release [8]. The main reason is that CS can produce highly protonated amine groups in an acidic environment. By attaching hydrophobic tails to the amino groups of CS, NPs prepared from hydrophobically modified CS can entrap hydrophobic anticancer drugs [9]. NPs prepared from hydrophobic CS with a higher degree of substitution show a higher degree of intracellular uptake from enhanced hydrophobic interactions with the cell membranes [10]. To prepare drug-loaded CS NPs, polyanionic cross-linking agents like sodium tripolyphosphate (TPP) can induce the sol–gel transition of CS by ionotropic gelation [11]. The mild processing conditions in an aqueous solution using non-toxic reagents and low energy inputs have made this method attractive in many biological applications, including oral drug delivery [12], protein delivery [13], oligonucleotide delivery [14], and plasmid DNA delivery [15]. However, even with this well-established protocol for preparing CS NPs, the influence of the processing parameters on the particle size or the drug release behavior of the NPs is not yet well established. Therefore, the key factors to optimize the processing parameters for CS/TPP cross-linking by the ionic gelation process and to minimize the size of the obtained NPs for controlled drug release are still missing.

Among the hydrophobic moieties that can be used to modify CS, oleic acid (OA) is a good choice [16]. Oleic acid, a major monounsaturated fatty acid found in olive oil, plays a role in activating several intracellular pathways during carcinoma development [17]. It has been reported that OA can induce apoptosis and autophagy for tongue squamous cell carcinoma treatment [18]. It also demonstrates an anti-tumor effect in hepatocellular carcinoma cell lines by reducing autophagy [19]. The anti-proliferative activities of OA are related to the PTEN/AKT/mTOR pathway in endometrial cancer [20]. Therefore, we modified CS with hydrophobic OA molecules to obtain oleoyl-chitosan (OC), which can entrap DOX through hydrophobic interactions. Furthermore, the drug-loaded NPs can provide pH-responsive controlled drug release due to the electrostatic repulsion between chitosan and DOX, allowing for effective drug release.

To develop smart, pH-sensitive magnetic NPs based on CS for targeted DOX delivery, OC was cross-linked with TPP to co-entrap DOX and Fe_3_O_4_, resulting in DOX-entrapped magnetic OC (DOX-MOC) NPs. Furthermore, we grafted FA to OC and used FA-OC to prepare magnetic FA-conjugated DOX-entrapped magnetic OC (FA-DOX-MOC) NPs to increase the targeting ability of the nanocarriers towards cancer cells. The FA-targeted and magnetic targeted NPs can accommodate DOX through hydrophobic interactions and regulate drug release with pH changes. These NPs may provide an efficient nanomedicine approach for cancer chemotherapy.

## 2. Materials and Methods

### 2.1. Materials

Chitosan (CS, molecular weight 110,000–190,000 Da, 85% degree of deacetylation), Fe (II) chloride tetrahydrate, Fe (III) chloride hexahydrate, doxorubicin hydrochloride (DOX), fluorescein isothiocyanate (FITC), folic acid (FA), Dulbecco’s modified Eagle’s medium-low glucose (DMEM), fetal bovine serum (FBS), and 3-[4,5-dimethylthiazol-2-yl]-2,5-diphenyl tetrazolium bromide (MTT) were purchased from Sigma-Aldrich (St. Louis, MO, USA). 1-Ethyl-3-(3-dimethylaminopropyl) carbodiimide (EDC), 4′,6-diamidino-2-phenylindole (DAPI), and a Live/Dead viability/cytotoxicity kit were purchased from Thermo Fisher Scientific (Waltham, MA, USA). All chemicals used were of analytical grade and were used as received without any further purification.

### 2.2. Preparation of Superparamagnetic Iron Oxide

We used a chemical co-precipitation method to prepare iron oxide (Fe_3_O_4_) magnetic NPs [21]. During the reaction, an alkaline solution was added to make the solution supersaturated, allowing iron ions to precipitate and form iron oxide NPs. In preparation, we first weighed 4.75 g and 1.75 g of FeCl_3_·6H_2_O and FeCl_2_·4H_2_O, respectively, and poured them into 80 mL of deionized water. To minimize oxygen exposure during the reaction, which would oxidize Fe_3_O_4_ into non-magnetic α-Fe_2_O_3_, we purged the solution with nitrogen beforehand and controlled the reaction temperature at 60 °C. After one hour of nitrogen gas purging, 8 mL of NH_4_OH solution was added while magnetically stirring at 600 rpm to change the solution pH to an alkaline value. The formation of NPs was indicated by the color change in the solution from yellow to black. After stirring for another 30 min to fully complete the reaction, we separated the NPs with a strong magnet and washed them with deionized water. The washing steps were repeated three to five times, and the particle suspension was dialyzed against distilled water at room temperature with a 3500 molecular weight cut-off membrane for 7 days.

### 2.3. Synthesis of Oleoyl-Chitosan (OC)

The amine groups of CS and the carboxyl groups of OA were used to form amide bonds after activating the carboxyl groups with EDC [22]. After dissolving 1 g CS in 1% acetic acid to make a 1% (*w*/*v*) CS solution, 85 mL of methanol was added. After dissolving 0.181 g OA and 0.1 g EDC in 15 mL methanol solution, the solution was added drop-wise to the CS solution and continuously stirred for 24 h. After the reaction, the reaction solution was poured into 200 mL of a methanol/ammonia solution (7:3) and stirred to precipitate OC. The precipitate was recovered by filtration, washed with distilled deionized water, and freeze-dried. The amount of OA in the OC was estimated from the change in the amine group in CS, as one OA molecule can react with one amine group in CS. The amine groups in CS were quantified using the phthalaldehyde (OPA) method [23]. The OA content in the OC was calculated to be 67.6 mg OA/g OC. The molar ratio of EDC (molecular weight = 155.2 g/mol) to OA (molecular weight = 281.5 g/mol) was fixed at 1:1 during the preparation of OC using 0.181 g OA and 0.1 g EDC.

### 2.4. Synthesis of Folic Acid-Conjugated Oleoyl-Chitosan (FA-OC)

First, 0.2 g of OC, as prepared above, was dissolved in 1% acetic acid solution to prepare a 1% (*w*/*v*) OC solution, and 17 mL of dimethyl sulfoxide (DMSO) was added. After dissolving 0.223 g FA and 0.09 g EDC in 3 mL DMSO, the solution was added drop-wise to the OC solution and stirred at room temperature for 24 h, protected from light. After the reaction, the reaction solution was poured into 40 mL DMSO/ammonia solution (7:3) to precipitate FA-OC. The FA-OC was finally recovered by filtration, washed with distilled deionized water, and freeze-dried to complete the preparation. The FA content was quantified by determining the solution absorbance at 368 nm in a 0.2 (*w*/*w*) FA-OC solution prepared in 3% acetic acid. A standard curve of FA was prepared from 0.01 to 0.5 mg/mL to calculate the concentration of FA in FA-OC.

### 2.5. Synthesis of Nanoparticles

**Scheme 1:**
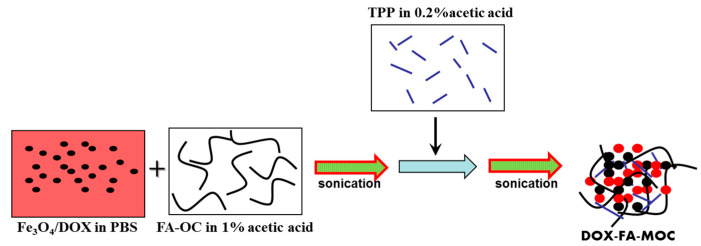
A schematic representation of the preparation process of doxorubicin (DOX)-loaded folic acid (FA)-conjugated magnetic oleoyl-chitosan (OC) (DOX-FA-MOC) nanoparticles.

Nanoparticles were synthesized from the OC or FA-OC solution in 1% acetic acid, TPP solution in 0.2% acetic acid, and Fe_3_O_4_/DOX solution in phosphate-buffered saline (PBS) at pH 7.4. The parameters studied during synthesis included OC:TPP mass ratios of 1:1, 1:2, and 1:4, and OC concentrations of 0.05, 0.1 and 0.2 (*w*/*w*). After adding 200 μL of a 20 mg/mL Fe_3_O_4_ solution and 200 μL of the OC or FA-OC solution to a 1.5 mL centrifuge tube, the solution was sonicated with a Misonix S4000 Ultrasonic Processor (Misonix, Faemingdale, NY, USA) at 600 W equipped with a cup horn for 5 min at 4 °C. Then, 200 μL of a 0.8% TPP solution was added, and the solution was sonicated at 4 °C for another 5 min for the formation of magnetic oleoyl-chitosan (MOC) or folic acid (FA)-conjugated MOC (FA-MOC) NPs. After synthesis, the NPs were separated using a magnet and washed twice with deionized water. For the synthesis of DOX-loaded NPs from MOC (DOX-MOC) or FA-MOC (DOX-FA-MOC), 200 μL of the 20 mg/mL Fe_3_O_4_/0.5 mg/mL DOX solution was used to replace the Fe_3_O_4_ solution, and similar steps were followed (Scheme 1).

### 2.6. Synthesis of Fluorescent Nanoparticles

To prepare fluorescent nanoparticles for tracking intracellular uptake, MOC or FA-MOC (5 mg/mL) was reacted with 2 mg/mL fluorescein isothiocyanate (FITC) in PBS at pH 7.4 for one hour to form covalent bonds between the isothiocyanate group in FITC and the primary amine group in OC. The NPs were washed three times with PBS to remove free FITC.

### 2.7. Drug Loading and Release

After encapsulation of DOX in FA-MOC, the DOX-FA-MOC was separated with a magnet, and the solution absorbance of the supernatant was measured at 490 nm. The concentration of the unencapsulated DOX was obtained from a calibration curve constructed for DOX. The encapsulation efficiency (EE) and loading efficiency (LE) were calculated from the following equations:

EE (%) = (Weight of loaded DOX/Weight of added DOX) × 100(1)

LE (%) = (Weight of loaded DOX (mg)/Weight of DOX-loaded NPs) × 100(2)

For drug release, 3 mg of DOX-FA-MOC NPs were dispersed in 0.6 mL of PBS at pH 5.5 or 7.4 and at 37 °C and shaken at 120 rpm in the dark. At predetermined times, the NPs were separated with a magnet, and the supernatant was analyzed for solution absorbance at 490 nm to demine the amount of released DOX. After completely removing the supernatant, an equal amount of PBS was added to continue the drug release experiment. The amount of released drug at each time point was added to calculate the cumulative drug release percentage using the following equation:

Drug release (%) = (Weight of released DOX/Weight of loaded DOX) × 100(3)

### 2.8. Characterization of Nanoparticles

The particle morphology was analyzed using transmission electron microscopy (TEM, JEOL JEM 2000EII, Tokyo, Japan). The particle size and zeta potential were assessed using dynamic light scattering (DLS, Zetasizer Nano ZS 90, Malvern Panalytical, Worcestershire, UK). A Siemens D5005 X-ray diffractometer (Siemens, Munich, Germany) with a CuK X-ray source (1.54 A°) from 10° to 70° was used for X-ray diffraction (XRD) analysis. After mixing NP powder with KBr, a Horiba (FT-730) spectrophotometer (Horiba, Kyoto, Japan) was used to perform Fourier-transform infrared (FTIR) spectroscopy. The thermal decomposition properties were analyzed using thermogravimetric analysis (TGA) with a TGA 2050 analyzer from TA Instruments (New Castle, DE, USA) up to 700 °C at 10 °C/min in a nitrogen environment. The superparamagnetic characteristics were analyzed using a superconducting quantum interference device (SQUID, MPMS XL-7, Quantum Design, San Diego, CA, USA). The percentage of iron oxide in magnetic samples was calculated using an Agilent Varian 710-ES inductively coupled plasma optical emission spectrometer (ICP-OES) (Agilent, Santa Clara, CA, USA) based on the iron content.

### 2.9. Intracellular Uptake and Cytotoxicity

To observe the intracellular uptake by cancer cells, a sterilized 15-millimeter glass slide was placed in a well of a 24-well plate. A 200 μL cell solution containing 1 × 10^4^ U87 cells suspended in a cell culture medium (90% DMEM and 10% FBS) was added to the top of the slide for cell attachment. After 24 h, the culture medium was removed and replaced with 200 μL FITC-conjugated NP suspension (83.3 μg/mL). After one hour of incubation for intracellular uptake, the culture medium was removed, and cells in the well were washed with PBS and fixed with 4% paraformaldehyde for 15 min. After washing with PBS, the cell nucleus was stained with 1 μg/mL DAPI for 5 min, washed with PBS, and observed under a Zeiss LSM780 laser scanning confocal microscope (Zeiss, Oberkochen, Germany). The effect of FA modification on intracellular uptake was studied following similar steps but by saturating the folate receptors on the cell surface with 1 mg/mL FA for one hour [24].

For cytotoxicity assessment, 200 μL of a U87 cell suspension (2.5 × 10^5^ cell/mL) was added to each well in a 48-well cell culture plate and incubated for 24 h for cell attachment. After removing the cell culture medium, 200 μL fresh medium containing the tested NPs was added, and the cell culture was continued. At 24, 48, and 72 h of culture, the cell viability was measured using 3-[4,5-dimethylthiazol-2-yl]-2,5-diphenyltetrazolium bromide (MTT) assays. The cells were washed with PBS, supplemented with 200 μL PBS and 40 μL MTT reagent (5 mg/mL), and reacted for 3 h. After removing the solution in each well, 200 μL DMSO was added to each well, and the solution absorbance value was measured with an ELISA microplate reader at 570 nm. The relative cell viability (%) was calculated based on the absorbance values of cells cultured in the cell culture medium (taken as 100%). The DOX concentration was tested at 5, 7.5, 10, 25, 50, 75, 100, and 150 μg/mL for free or encapsulated drug, and the half-maximal inhibitory concentration (IC_50_) of the drug was determined.

### 2.10. Cell Viability by Live/Dead Staining

For direct observation of the cytotoxicity from the nanoparticles, a sterilized 15-millimeter glass slide was placed in a well of a 24-well plate, and 200 μL of a U87 cell suspension in a cell culture medium (90% DMEM and 10% FBS) containing 5 × 10^4^ cells was added to the top of the slide for cell attachment. After 24 h, the culture medium was removed and replaced with 200 μL of an NP suspension and reacted for 2 h. After removing the culture medium and washing the sample with PBS, the cells were stained with the Live/Dead viability/cytotoxicity kit for 30 min and observed under a Zeiss LSM780 laser scanning confocal microscope. Calcein AM (Ex_488_/Em_518_) dye can stain live cells with green fluorescence, and propidium iodide (Ex_488_/Em_615_) dye can stain dead cells with red fluorescence. For magnetic targeting, a magnetic field was generated by fixing a 4 mm diameter permanent magnet (2800 Gauss) to the bottom of each well. Overall, 5 × 10^4^ U87 cells were cultured for 24 h in each well and treated with the NP suspension for 2 h before Live/Dead staining.

### 2.11. Statistical Analysis

The data are reported as the mean ± standard deviation (SD). A one-way analysis of variance (ANOVA) with *p* < 0.05 was considered statistically significant.

## 3. Results and Discussion

### 3.1. Preparation of Nanoparticles

To achieve an efficient drug delivery system for cancer treatment, the size of drug-loaded NPs is usually controlled to be below 400 nm [25]. With the enhanced permeability and retention (EPR) effect, NPs can permeate through vessels in the tumor tissue and be retained in the tumor bed due to reduced lymphatic drainage [26]. Therefore, we aimed to reduce the size of the NPs by controlling the parameters used during the synthesis step. We also aimed to prepare pH-responsive NPs with a high drug release rate in the low-pH endosomal environment to prevent premature extracellular drug release. Therefore, several parameters were studied regarding their effects on the properties of NPs, including the OC:TPP mass ratio (1:1, 1:2, and 1:4) and OC concentration (0.05%, 01%, and 0.2%). There are many examples of CS modified by hydrophobic molecules, such as deoxycholic acid, linolenic acid, oleic acid, etc. [27,28,29,30]. In general, modification with hydrophobic moieties is conducive to self-assembling the materials into nanoscale carriers and shows entrapment of hydrophobic drugs. Although some of the amine groups of CS are replaced by OA to increase the hydrophobicity of OC, the remaining positively charged amine groups can provide electrostatic interactions with the three phosphate groups of TPP. This leads to cross-linking between OC and TPP and forms MOC NPs by electrostatic attraction [31,32,33]. Due to the electrostatic repulsion between chains, OC usually assumes an extended configuration in acidic solutions. By intermolecular cross-linking reactions, OC spontaneously changes its original extended configuration to a tight conformation in the NPs after adding TPP [34]. As interactions between OC and TPP can influence the formation of the drug-loaded NPs by cross-linking, we studied the effect of the OC:TPP mass ratio first.

Using 0.1% OC, when the OC:TPP mass ratio changed from 1:1 to 1:4, the particle size of DOX-MOC NPs increased, but the zeta potential decreased (Table 1). By mixing more TPP with OC, additional cross-linking between OC and TPP is expected. This leads to more negatively charged TPP molecules being incorporated in the NPs and increases the particle size but decreases the zeta potential. The EE and LE also increased with increasing particle size. Nonetheless, it should be noted that the pKa of DOX is 8.5, and the drug will carry a positive charge under acidic preparation conditions. The repulsion between OC and DOX may influence DOX encapsulation [35].

**Figure 1:**
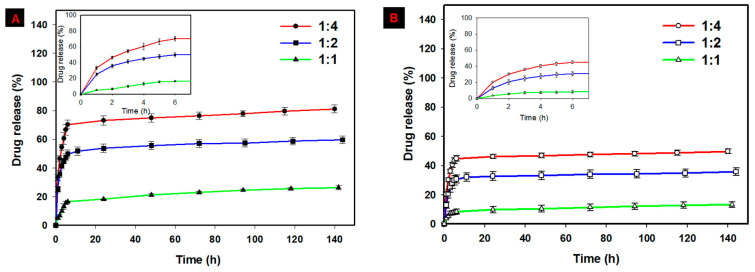
The effects of OC:TPP mass ratio on drug release in vitro at pH 5.5 (**A**) and 7.4 (**B**). OC concentration = 0.1%.

The DOX release was studied in neutral and acidic pH values to simulate the physiological and endosomal pH values by placing the DOX-MOC NPs in PBS at pH 5.5 and pH 7.4 at 37 °C (Figure 1). From the release curve, it was found that the release of the drug followed a two-stage process. A fast-release behavior was observed in the initial stage, followed by a second stage of slower release. During the preparation of the NPs, the DOX was first in contact with Fe_3_O_4_ magnetic nanoparticles and dispersed in OC, followed by TPP cross-linking. The presence of Fe_3_O_4_ NPs may lead to the adsorption of DOX to its surface, which also affects the DOX release from the NPs. This phenomenon makes the DOX release appear to follow the drug release profile of multi-layer coated carrier dosage forms [36]. In this dosage form, since the drug is dispersed in both inner and outer layers, the drug in the outer layer will be released first. At the later stage, a stable drug release rate will be observed when the drug in the inner layer starts to be released in the second phase with a zero-order release profile [37,38,39]. The drug release curve shows that the NPs released more DOX at pH 5.5 than pH 7.4. At pH 5.5, OC is highly protonated, and the repulsive force between OC molecules reduces the degree of cross-linking between OC and TPP compared to pH 7.4. The dissolution of DOX-MOC is also faster at pH 5.4 than at pH 7.4, meaning that DOX can be released by diffusing through the loose polymeric network in the NPs. This results in a higher release rate and a higher release percentage of DOX. The OC:TPP mass ratio affects the drug release behavior; more TPP results in a higher drug release rate and drug release percentage at both pH values. Choosing an OC:TPP mass ratio of 1:4 to achieve the best therapeutic effect, the DOX-MOC NPs can release ~71% of the drug in 5 h at pH 5.5 vs. ~45% at pH 7.4.

Regarding the effect of OC concentration, higher OC concentrations increased the particle size and zeta potential of DOX-MOC NPs (Table 2). Using more OC in the preparation step can lead to more polymer being cross-linked by TPP, forming larger NPs with a higher zeta potential. This coincides with increased EE and LE values.

**Figure 2:**
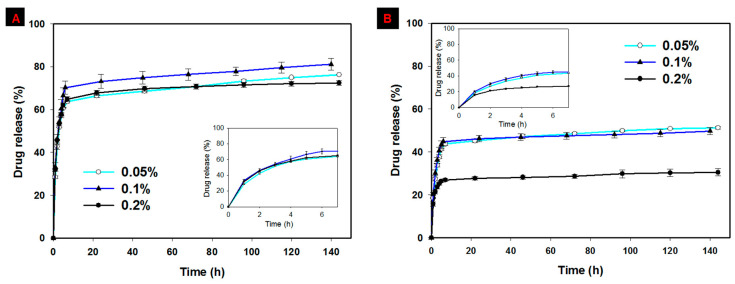
The effects of OC concentration on drug release in vitro at pH 5.5 (**A**) and 7.4 (**B**). OC:TPP mass ratio = 1:4.

NPs prepared with different OC concentrations were used for in vitro drug release. At pH 5.5, the effect of OC concentrations on the release curve was insignificant (Figure 2). Nonetheless, NPs prepared with 0.2% OC showed lower cumulative drug release than the other two concentrations at pH 7.4. At an acidic pH value, OC is highly positively charged, which allows DOX to be released from the NPs irrespective of its concentration. At pH 7.4, OC is less charged, making NPs made from 0.2% OC more resistant to drug release. Considering the difference in pH-sensitive drug release behavior, the 0.2% OC concentration was selected as the optimal preparation condition to prepare drug-loaded NPs.

### 3.2. Characterization of Nanoparticles

For FA-modified NPs, we chose to prepare FA-OC and developed FA-MOC NPs from this polymer instead of grafting FA to MOC NPs to avoid release of the drug during FA conjugation. EDC was used to activate the carboxyl groups in FA for subsequent reaction with the amine groups in OC to synthesize FA-OC. The FA content in FA-OC was calculated to be 68.6 mg FA/g FA-OC, and FA-OC can be dissolved in acetic acid. The particle size, zeta potential, LE, and EE of MOC NPs, DOX-MOC NPs, and FA-DOX-MOC NPs are summarized in Table 3. There is no significant difference in particle size among all NPs. The FA-DOX-MOC NPs showed insignificant differences in particle size, EE, and LE compared to DOX-MOC NPs, indicating that the condition chosen for DOX-MOC preparation could also be adopted for FA-DOX-MOC. The only significant difference noted is the zeta potential change due to the consumption of positively charged amine groups in OC during the synthesis of FA-OC. This decreased the zeta potential of FA-DOX-MOC NPs compared with DOX-MOC NPs. The entrapment of positively charged DOX increased the zeta potential of DOX-MOC NPs compared to MOC NPs.

**Figure 3:**
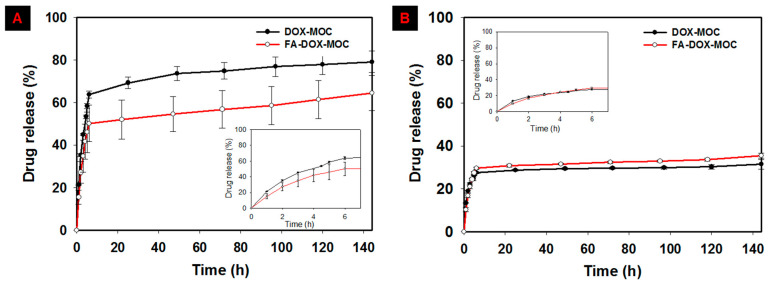
The drug release curves at pH 5.5 (**A**) and 7.4 (**B**) for FA-DOX-MOC and DOX-MOC NPs.

The drug release curves for FA-DOX-MOC and DOX-MOC NPs are compared in Figure 3. At pH 7.4, FA grafting did not affect the release kinetics and release percentage. In contrast, the drug release percentage decreased from 80% for DOX-MOC NPs to 60% for FA-DOX-MOC NPs at pH 5.5. This may arise from the difference in surface potential between the NPs. As the surface of FA-DOX-MOC becomes more negatively charged in acidic conditions, the release of positively charged DOX will be hindered. Using the Korsmeyer–Peppas model for drug release from a swellable polymeric matrix to the first 60% of drug release, the release kinetics data during the first 5 h were fitted with the following equation [40]:

(4) $$\frac{M_{t}}{M_{\infty }}=kt^{n}$$

where M_t_ is the cumulative amount of drug released at time t, M_∞_ is the cumulative amount of drug released after infinite time (144 h), M_t_/M_∞_ is the fraction of drug released at time t, k is the release rate constant, and n is the diffusion exponent. The value of n characterizes the release mechanism as Fickian diffusion or non-Fickian diffusion. For spherical NPs, n = 0.43 corresponds to Fickian diffusion, and the rate of solvent penetration is much slower than the rate of polymer chain relaxation for a diffusion-controlled system; 0.43 < n < 0.85 indicates anomalous (non-Fickian) diffusion, and the diffusion and polymer chain relaxation rates are comparable [41].

As shown in Table 4, the drug release kinetics can be fitted well with Equation (4) in all cases with R^2^ (coefficient of determination) = 0.99. The rate constant k was higher at pH 5.5 than at pH 7.4, indicating pH-responsive drug release with faster drug release in an acidic environment. The release mechanism is mostly anomalous due to swelling of the NPs, which can influence drug diffusion. However, DOX-MOC NPs at pH 7.4 showed a much smaller n-value (0.410), which is close to the Fickian-type drug release, indicating limited swelling of these NPs at neutral pH.

**Figure 4:**
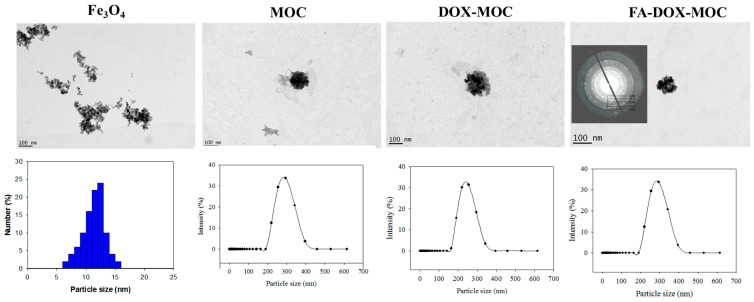
The transmission electron microscope (TEM) images and particle size distribution of Fe_3_O_4_ in MOC, DOX-MOC, and FA-DOX-MOC nanoparticles (bar = 100 nm). The particle size distribution is shown below the TEM image, determined by counting the size of discrete particles for Fe_3_O_4_, while it was determined by dynamic light scattering (DLS) for MOC, DOX-MOC, and FA-DOX-MOC NPs. The insert in the TEM image of FA-DOX-MOC is the selected area electron diffraction (SAED) pattern of the dark Fe_3_O_4_ nanoparticles.

The morphology of the NPs was observed using a transmission electron microscope. Figure 4 shows agglomerates of Fe_3_O_4_ formed by discrete nanoparticles with a mean particle size of 11.3 nm. The MOC, DOX-MOC, and FA-DOX-MOC NPs show similar spherical morphologies and particle sizes within 100 to 150 nm. These values are smaller than the particle size of ~250 nm estimated from dynamic light scattering (DLS) analysis. This difference exists between dried TEM samples with collapsed polymer chains and DLS samples with dispersed nanoparticles in aqueous solution [42]. The sample was negatively stained with phosphotungstic acid (PTA) to show darker Fe_3_O_4_ NPs entrapped within the brighter background of polymer chains [43]. In addition, the selected area electron diffraction (SAED) pattern of FA-DOX-MOC NPs also confirmed the presence of crystalline Fe_3_O_4_ [44]. The dense population of Fe_3_O_4_ within the NPs from the TEM image was confirmed by directly determining the Fe_3_O_4_ weight fraction in each sample using ion-coupled plasma optical emission spectrometry (ICP-OES), with weight fractions of 86.1%, 87.9%, and 84.7% for MOC, DOX-MOC, and FA-DOX-MOC, respectively.

**Figure 5:**
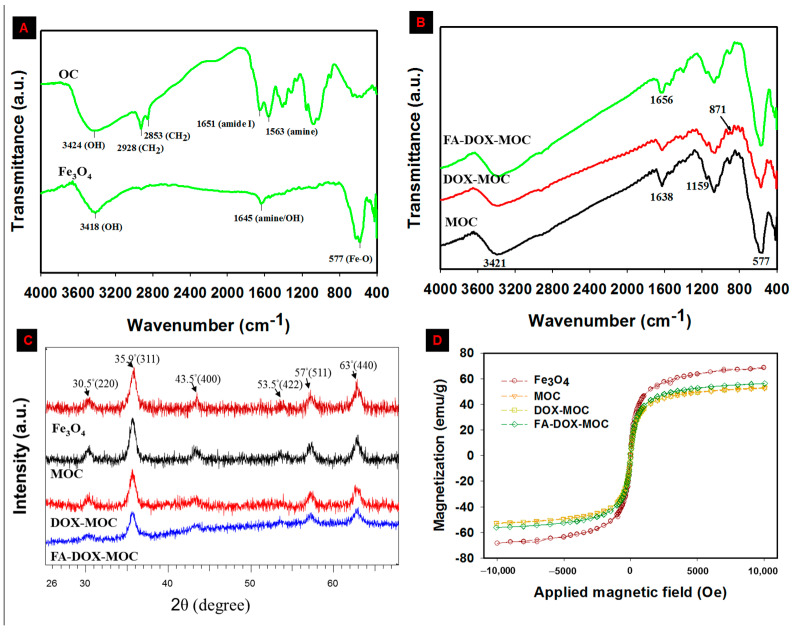
Fourier-transform infrared (FTIR) spectroscopy (**A**,**B**), X-ray diffraction (XRD) (**C**), and superconducting quantum interference device (SQUID) (**D**) analyses.

From the FTIR spectra in Figure 5A, OC shows characteristic peaks at 3424 cm^−1^ (OH), 2928 cm^−1^ (CH_2_), 2853 cm^−1^ (CH_2_), 1638 cm^−1^ (amide I), and 1563 cm^−1^ (amine). Fe_3_O_4_ shows characteristic peaks at 3418 cm^−1^ (-OH), 1645 cm^−1^ (amine or -OH), and 577 cm^−1^ (Fe-O). MOC, DOX-MOC, and FA-DOX-MOC show the characteristic peaks associated with OC and Fe_3_O_4_ (Figure 5B). In addition, the characteristic peak at 1159 cm^−1^, which is associated with the P = O or P-O-functional groups in TPP, can confirm the cross-linking reaction between TPP and OC. DOX-MOC shows a peak at 871 cm^−1^ (N-H) assigned to DOX, while other peaks at 1530 cm^−1^ (amide I) and 810 cm^−1^ (C-O-CH_3_) coincide with those in OC [45]. For FA-DOX-MOC, the characteristic peak at 1690 cm^−1^ from carboxylic acid groups in FA merges with the amide I peak and shows a broad peak at 1656 cm^−1^ [46].

The XRD analysis confirmed the crystalline structure of Fe_3_O_4_, with characteristic diffraction peaks at 2θ of 30.5° (220), 35.9° (311), 43.5° (400), 53.5° (422), 57° (511), and 63° (440) (Figure 5C) [47]. The blending of OC, FA-OC, TPP, or DOX did not change the Fe_3_O_4_ crystal structure from the XRD patterns of MOC, DOX-MOC, and FA-DOX-MOC. The Fe_3_O_4_ grain sizes calculated from the half-width of the highest peak at 35.9° with the Debye–Scherrer equation were 12.3, 12.3, 12.9, and 12.8 nm for Fe_3_O_4_, MOC, DOX-MOC, and FA-DOX-MOC, respectively. The ~12 nm particle size of Fe_3_O_4_ is consistent with that from TEM observation. The magnetization curves from the superconducting quantum interference device (SQUID) show hysteresis loops passing through the origin (Figure 5D). The residual (remnant) magnetization without a magnetic field was close to zero for all tested samples, indicating their superparamagnetic properties from entrapped Fe_3_O_4_. Superparamagnetic behavior depends on the particle size and occurred only for NPs less than ~20 nm in size [48]. This observation is consistent with the Fe_3_O_4_ particle size determined from TEM and XRD. The saturation magnetization for Fe_3_O_4_ was 68.7 emu/g, which is higher than the values for MOC (52.7 emu/g), DOX-MOC (53.1 emu/g), and FA-DOX-MOC (56.3 emu/g). This is due to the reduced Fe_3_O_4_ weight percentage in the NPs.

**Figure 6:**
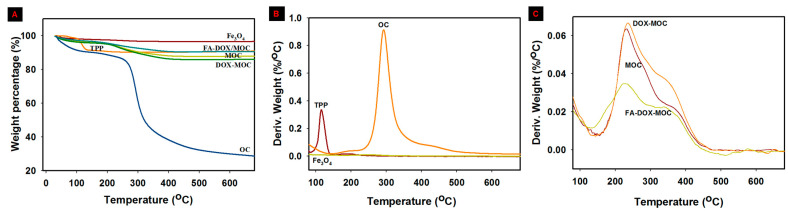
The thermogravimetric analysis (TGA) curves (**A**) and derivative thermogravimetric (DTG) curves (**B**,**C**).

The thermal decomposition was analyzed using thermogravimetric analysis (TGA) (Figure 6A). Weight loss before 100 °C was due to residual moisture and occurred only for OC. Fe_3_O_4_ was thermally stable up to 700 °C, with no mass change. TPP showed weight loss at 100–200 °C. For OC, a second stage of weight loss occurred between 200 and 400 °C, within which MOC, DOX-MOC, and FA-DOX-MOC also displayed thermal decomposition. The derivative thermogravimetric (DTG) curves reveal peak decomposition temperatures at 115 and 293 °C for TPP and OC, respectively (Figure 6B). For MOC, DOX-MOC, and FA-DOX-MOC, a single peak decomposition temperature is shown at 230 °C (Figure 6C). This may arise from the thermal decomposition of the cross-linked OC/TPP polymer chain and supports the expected mechanism of forming NPs by cross-linking OC (or FA-OC) with TPP.

### 3.3. Cell Culture Studies

**Figure 7:**
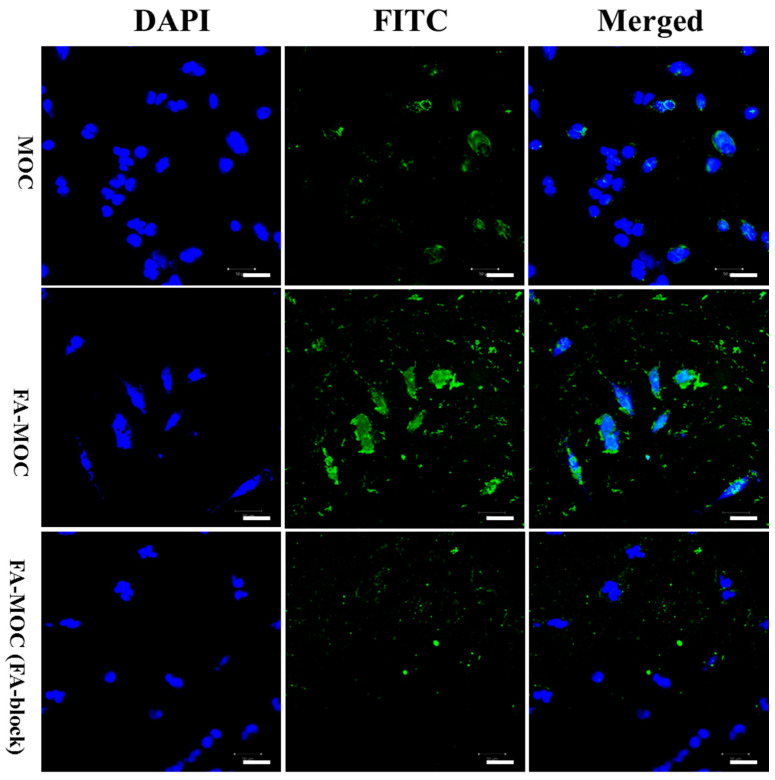
The intracellular uptake of FITC-labeled MOC or FA-MOC by U87 cells was examined by confocal microscopy. The cell nuclei were labeled with DAPI to show blue fluorescence. The green fluorescence is the FITC-labeled MOC or FA-MOC. Bar = 50 μm. The FA-block group used excess free FA to treat U87 cells for one hour before adding FITC-labeled FA-MOC.

To confirm that FA may increase intracellular uptake, we treated U87 cells with FITC-labeled MOC and FA-MOC for 3 h and observed the cells under a confocal microscope (Figure 7). The green fluorescence signal from FITC represents the location of the NPs, while the blue fluorescence is the nuclei counterstained with DAPI. The role FA plays in binding with overexpressed folate receptors on the surface of U87 cells was confirmed by pre-treating cells with excess FA for one hour before contacting them with FA-MOC in the FA-block group. Compared with MOC, FA-MOC could significantly increase the uptake of NPs, as indicated by the much higher green fluorescence signal from aggregated NPs around the cell nuclei. This FA-mediated effect can be confirmed from the FA-block group, where the green fluorescence signal intensity was significantly reduced to a level comparable to that in the MOC group. Undoubtedly, this is due to the saturation of folate receptors on the cell surface with FA, which hinders the binding of FA-MOC to the cell surface and intracellular uptake by endocytosis.

**Figure 8:**
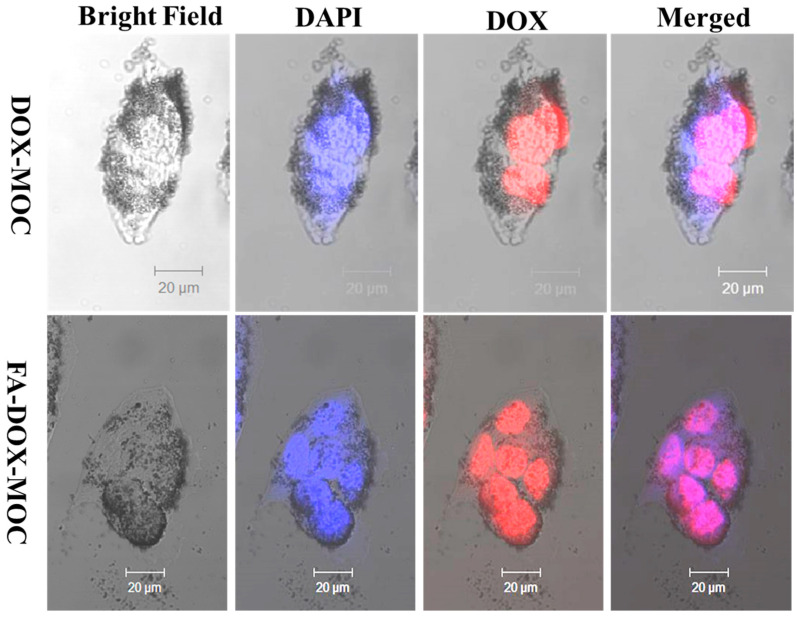
The localization of intracellular DOX by confocal microscopy 3 h after contacting U87 cells with DOX-MOC or FA-DOX-MC NPs. The cell nuclei were labeled with DAPI to show blue fluorescence. The red fluorescence is DOX. Bar = 20 μm.

Since DOX emits red fluorescence at 571 nm after excitation, the localization of DOX-loaded NPs after intracellular uptake was also studied using confocal microscopy. When DOX-MOC or DOX-FA-MOC NPs are engulfed by cancer cells, the NPs will stay in the cytoplasm, while released DOX will enter the cell nucleus to exert its cytotoxicity by chelating with DNA. This can be observed in Figure 8, which shows blue fluorescence from DAPI overlapping with red fluorescence from DOX in the merged images. However, the FA-DOX-MOC NP treatment appeared to provide more DOX in the nucleus than DOX-MOC NPs. This is consistent with the higher cellular uptake rate of FA-MOC shown in Figure 7.

**Figure 9:**
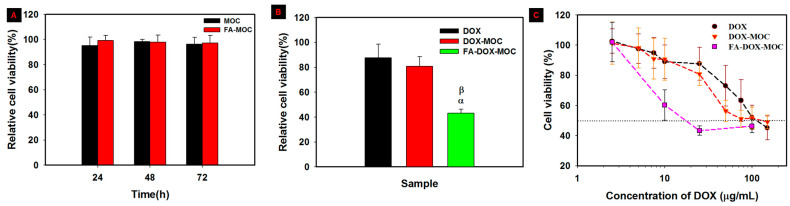
(**A**) The biocompatibility of MOC NPs and FA-MOC NPs (concentration = 83.3 μg/mL) was determined at different cell culture times. (**B**) The in vitro cytotoxicity of DOX and FA-DOX-MOC NPs (concentration of DOX = 25 μg/mL) was determined at 24 h cell culture time. ^α^ *p* < 0.05 compared with DOX; ^β^ *p* < 0.05 compared with DOX-MOC. (**C**) The effect of DOX concentration on the in vitro cytotoxicity of DOX, DOX-MOC, and FA-DOX-MOC was determined at 24 h cell culture time. The dash line is 50% cell viability for calculating IC_50_.

The biocompatibility of drug-free NPs was tested by culturing MOC or FA-MOC NPs with U87 cells. MTT assays were used to measure the relative cell viability (Figure 9A). Irrespective of cell culture times, neither MOC nor FA-MOC NPs exerted any cytotoxicity, as demonstrated by normalizing the MTT absorbance to the absorbance when cells were cultured in a cell medium without NPs at each time point (taken as 100%). To assess the cytotoxicity exerted by DOX-loaded NPs, U87 cells were treated with free DOX, DOX-MOC NPs, or FA-DOX-MOC NPs for 24 h at the same drug dosage (25 μg/mL), and the relative cell viability was determined using MTT assays. As shown in Figure 9B, there was no significant difference in relative cell viability between DOX-MOC and DOX, but FA-DOX-MOC significantly reduced cell survival compared with both groups. The mean cell survival rates were 80.8% and 87.7% for DOX-MOC and DOX, respectively. However, FA-DOX-MOC exerted significantly higher cytotoxicity toward U87 cancer cells, with a mean cell survival rate of 43.3%. This supports the use of FA for targeted delivery of DOX in cancer therapy. Subsequently, the drug was tested at different concentrations for its cytotoxicity to calculate the IC_50_ value, which is the drug concentration that can kill 50% of cancer cells in vitro. As shown in Figure 9C, the cell viability decreased with increasing DOX dosage. Nonetheless, the FA-DOX-MOC treatment showed the most pronounced decrease in cell viability. The IC_50_ of FA-DOX-MOC NPs was 17.3 µg/mL, which is 13% of that of DOX-MOC NPs (IC_50_ = 131.1 µg/mL) and 15% of that of DOX (IC_50_ = 114.9 µg/mL).

**Figure 10:**
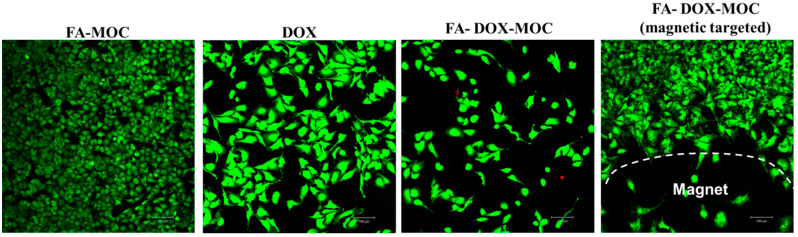
The Live/Dead staining of U87 cells after cell culture with FA-MOC, DOX (25 μg/mL), FA-DOX-MOC (25 μg/mL DOX). The FA-DOX-MOC (magnetic targeted) group included cell culture with FA-DOX-MOC (25 μg/mL DOX) in the presence of a magnetic field created by placing a magnet at the bottom of the well. Bar = 100 μm. Live cells emit green fluorescence and dead cells emit red fluorescence.

The preferability of FA-DOX-MOC over DOX for magnetic guided cancer therapy is supported by the Live/Dead staining results. As shown in Figure 10, the drug-loaded FA-DOX-MOC NPs resulted in much fewer viable cells emitting a green fluorescence signal compared to those treated with drug-free FA-MOC NPs. The cancer-cell-killing ability was also much higher for FA-DOX-MOC than DOX at the same drug dosage (25 μg/mL DOX). This is consistent with its higher cytotoxicity observed in the MTT assays shown in Figure 9C. Most importantly, when applying a magnetic field during cell culture by placing a magnet at the bottom of the well, FA-DOX-MOC NPs could be magnetically guided by the magnet for magnetic targeting of the cancer cells. Fewer live cells in the magnetic target zone (within the dotted line in Figure 10) were found; in contrast, live cells comparable in number to the control FA-MOC group were found in the surrounding non-targeted area. Taken together, the magnetic targeting effect offered by FA-DOX-MOC can augment the ligand-targeting effect from FA. The NPs will be restricted to a specific area by a magnetic field for enhanced intracellular uptake by U87 cells mediated by FA, which can increase the intracellular concentration of DOX for killing the cancer cells while minimizing cytotoxicity towards neighboring areas not targeted by the magnetic field. The dual-targeting capability of FA-DOX-MOC NPs is expected to provide an efficient nanomedicine approach for chemotherapy in vivo with minimum side effects [49].

## 4. Conclusions

We used TPP to cross-link OC or FA-OC for the co-entrapment of Fe_3_O_4_ and DOX in a pH-sensitive smart drug delivery system for targeted cancer therapy. The optimized conditions for preparing the NPs used an OCS:TPP mass ratio of 1:4 and an OC concentration of 0.2%. The synthesized DOX-OC and FA-DOX-OC NPs were spherical in shape, with a particle size of ~250 nm, ~83% EE, and ~2.8% LE of DOX. The DOX release was pH-sensitive, with faster drug release at pH 5.5 than at pH 7.4, which can facilitate intracellular drug release in an acidic endosomal environment. The initial drug release profile could be modeled satisfactorily with the Korsmeyer–Peppas equation. FA could enhance the intracellular uptake of the NPs and DOX accumulation in the cell nuclei, providing significantly higher cytotoxicity toward U87 cancer cells. The IC_50_ of FA-DOX-MOC NPs reduced to 13% of that of DOX-MOC and 15% of that of DOX. With an 85% Fe_3_O_4_ content and 56.3 emu/g saturation magnetization, the FA-DOX-MOC NPs could be guided with a magnetic field for magnetically targeted drug delivery in cancer therapy.

## Figures and Tables

**Table 1:** The particle size, zeta potential, EE, and LE of DOX-MOC NPs prepared with different OC:TPP mass ratios. OC concentration = 0.1%.

OCS:TPP Mass Ratio	Particle Size (nm)	Zeta Potential (mV)	EE (%) *	LE (%) *
1:1	212.3 ± 3.4	23.0 ± 0.5	56.6 ± 2.4	1.9 ± 0.1
1:2	227.3 ± 8.5	7.9 ± 0.3	77.1 ± 1.9	2.3 ± 0.1
1:4	242.2 ± 3.8	3.9 ± 0.4	78.8 ± 2.9	2.4 ± 0.2

* EE: DOX encapsulation efficiency; LE: DOX loading efficiency.

**Table 2:** The particle size, zeta potential, EE, and LE of DOX-MOC NPs prepared with different OC concentrations.

OC Concentration (%)	Particle Size (nm)	Zeta Potential (mV)	EE (%) *	LE (%) *
0.05	209.8 ± 4.3	−7.6 ± 0.3	70.9 ± 1.7	1.6 ± 0.1
0.1	242.2 ± 3.8	3.9 ± 0.2	78.8 ± 2.9	2.4 ± 0.2
0.2	257.1 ± 8.5	11.4 ± 0.1	82.4 ± 2.5	2.8 ± 0.1

* EE: DOX encapsulation efficiency; LE: DOX loading efficiency.

**Table 3:** The particle size, polydispersity index, zeta potential, EE, and LE of different NPs.

Nanoparticle	Particle Size (nm)	PDI	Zeta Potential (mV)	EE (%) *	LE (%) *
MOC	251.3 ± 4.7	0.23 ± 0.03	6.4 ± 0.2	-	-
DOX-MOC	257.1 ± 18.5	0.19 ± 0.02	11.4 ± 0.1	83.1 ± 3.9	2.81 ± 0.17
FA-DOX-MOC	246.0 ± 14.0	0.21 ± 0.02	−13.8 ± 0.3	83.9 ± 2.9	2.83 ± 0.11

* EE: DOX encapsulation efficiency; LE: DOX loading efficiency; PDI: polydispersity index.

**Table 4:** The release kinetics parameters of DOX from DOX-MOC and FA-DOX-MOC nanoparticles in the Korsmeyer–Peppas model for drug release *.

Nanoparticle	pH	n	k	R^2^
DOX-MOC	5.5	0.608	22.3	0.99
	7.4	0.410	13.8	0.99
FA-DOX-MOC	5.5	0.653	17.0	0.99
	7.4	0.592	10.6	0.99

* The data in Figure 3 during the first 5 h are fitted with the equation $\frac{M_{t}}{M_{\infty }}=kt^{n}.$

## Data Availability

Data are contained within the article.
